# Gene-based partial least-squares approaches for detecting rare variant associations with complex traits

**DOI:** 10.1186/1753-6561-5-S9-S19

**Published:** 2011-11-29

**Authors:** Asuman S Turkmen, Shili Lin

**Affiliations:** 1 Department of Statistics, The Ohio State University, 1179 University Drive, Newark, OH 43055, USA; 2 Department of Statistics, The Ohio State University, 1958 Neil Avenue, 404 Cockins Columbus, OH 43210, USA

## Abstract

Genome-wide association studies are largely based on single-nucleotide polymorphisms and rest on the common disease/common variants (single-nucleotide polymorphisms) hypothesis. However, it has been argued in the last few years and is well accepted now that rare variants are valuable for studying common diseases. Although current genome-wide association studies have successfully discovered many genetic variants that are associated with common diseases, detecting associated rare variants remains a great challenge. Here, we propose two partial least-squares approaches to aggregate the signals of many single-nucleotide polymorphisms (SNPs) within a gene to reveal possible genetic effects related to rare variants. The availability of the 1000 Genomes Project offers us the opportunity to evaluate the effectiveness of these two gene-based approaches. Compared to results from a SNP-based analysis, the proposed methods were able to identify some (rare) SNPs that were missed by the SNP-based analysis.

## Background

The past decade has seen a surge of interest in genome-wide association studies because of the availability of densely situated single-nucleotide polymorphisms (SNPs) throughout the genome and because of new exciting results that offer tremendous hope and optimism [1]. However, such hope and optimism have been dampened by the limited success of reaping benefits from the discoveries, because the identified SNPs contribute little to the explanation of the underlying variability. Such realizations set off vigorous debates, including those in the popular media. However, second-generation sequencing technology has made it practically feasible to reliably genotype rare SNP variants.

In mapping genes that contribute to common diseases, a popular hypothesis is that causal variants are common in the population. However, it is now hypothesized that complex traits may be caused by the combined contribution of many rare variants (minor allele frequency [MAF] < 0.05); this contribution is one of the many potential explanations for the missing heritability [2]. However, even though rare variants can now be genotyped efficiently and accurately using second-generation sequencing technology, detecting associations between rare variants and disease using SNP-based methods is frequently ineffective, especially when the effect size is small. Therefore there is a pressing need for statistical methods that can detect rare variants.

Here, we propose a gene-based partial least-squares (GBPLS) method for detecting associations with quantitative traits. By considering a gene as the fundamental unit in our modeling, we hope that this aggregation effect will help us uncover associations that are too weak to be detected for individual SNPs. To reduce the computational burden and noise caused by the inclusion of a large number of noncausal variants, we use a screening procedure that selects the top genes, to which we apply the proposed methods.

Our data analysis was performed with the knowledge of the answers, which contributed to our focus on gene-based strategies, although the proposed method does not make use of the specific simulation model. As such, we believe that the proposed methods would be suitable for the analysis of real data because SNPs within genes are often working together to regulate phenotypes.

## Methods

### Data

The Genetic Analysis Workshop 17 (GAW17) data contain one family data set and one population data set. Two hundred replicates of the trait simulation were carried out in both data sets [3]. In this study, we used the population data consisting of a collection of 697 unrelated individuals. Information for each individual in each replicate includes the genotypes of 24,487 SNPs in 3,205 genes for each individual, covariates Age, Sex, and Smoke, quantitative traits Q_1_, Q_2_, Q_4_, and qualitative trait Affected. Note that the SNP genotypes are held fixed for all 200 replicates. Of the 24,487 SNPs, almost 91% and 75% have a MAF less than 0.1 and 0.01, respectively. We carried out our proposed procedures for two quantitative traits: Q_1_ and Q_2_. The design matrix of the genotype *X* of size *n x p* is constructed by labeling genotypes *AA*, *Aa*, and *aa* as 0, 1, and 2, respectively, where, *n* = 697 and *p* = 24,487. All 200 replicates are used to learn about association. To control population structure, we also used in the analyses the non-SNP covariates Age, Sex, and Smoke and the first 10 principal components (PCs) *P_k_* (*k* = 1, 2, …, 10) of genotype data.

### Data preprocessing: the screening stage

The screening stage is composed of two steps. In the first step, correlations between the 24,487 SNPs and the quantitative trait *Q_j_* (*j* = 1, 2), are calculated. Each gene is scored by the largest absolute value of the correlation between the trait and the SNPs within the corresponding gene. We then order the 3,205 genes based on these scores and retain only the top 1,000 to reduce the amount of computation in subsequent analyses. In the second step, we randomly assign all of the SNPs in the top 1,000 genes into the same set of 1,000 genes 500 times to control the different gene sizes. We recompute the gene scores for each randomization and calculate the *p*-values as the proportion of recalculated gene scores that are as extreme as or more extreme than the original gene scores. We rank the 1,000 genes based on the *p*-values (i.e., the smaller the *p*-value, the smaller the rank of the gene). The output of this stage produces the ordered list of the top 1,000 genes.

With the knowledge of the simulated model, we can see that this preliminary screening stage retains most of the genes involved in the trait and excludes many noncausal genes. We ran the analysis for values different from 1,000 as well and concluded that the top 1,000 yields the best results. Because of space limitations, we report here the results based on only the top 1,000 genes. We use  to denote the lower dimensional design matrix *X*, where the columns of  are constructed by the SNPs within the first *t* of the 1,000 ordered genes, where *t* = 1, 2, …, 1,000. Similarly, the submatrix that stores only the SNPs within the gene *i* is denoted  for *i* = 1, 2, …, *t*. Although the initial screening stage helps to reduce the dimensionality of the design matrix greatly, there is still the curse of dimensionality problem because there are more SNPs than observations.

### Gene-based partial least-squares approach

**Figure 1:**
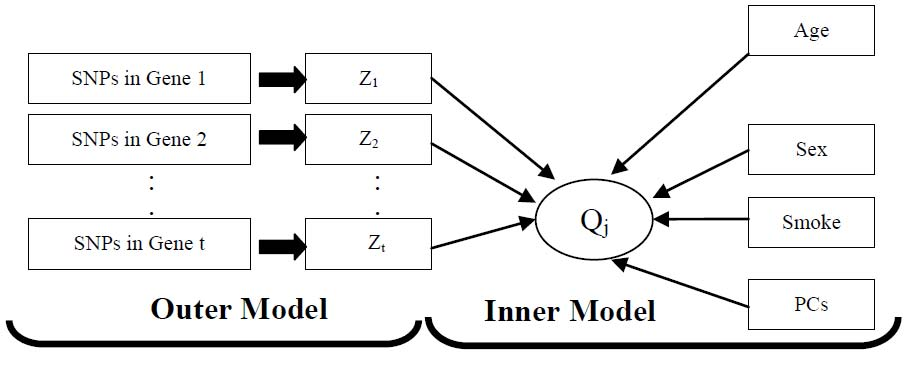
The GBPLS algorithm for *Q_j_* (*j* = 1, 2).

The general idea used in the proposed GBPLS approach can be summarized using the diagram in Figure 1. The approach specifies two sets of relationships: (1) the outer model, which links the SNPs within a given gene with a latent variable (LV; the *Z* variables in Figure 1) by simply aggregating them through a projection; and (2) the inner model, which specifies the relationships between predictors (LVs, non-SNP covariates, and the first 10 PCs) and the trait *Q_j_* (*j* = 1, 2). The coefficients corresponding to the outer and inner models are called outer and inner coefficients, respectively. We consider two specific GBPLS algorithms, GBPLS1 and GBPLS2. Both algorithms consists of stage 1 and 2, corresponding to the calculations of the outer and inner weights, respectively. However, they differ by the approaches used for the calculation, as we describe in the following.

In the GBPLS1 algorithm, partial least-squares path modeling (PLSPM) is used to calculate the outer coefficients. PLSPM is a statistical method that was developed for the analysis of structural equation models with latent variables. A formal presentation of the partial least-squares approach to latent variable path models is given by Wold [4]; a more recent reference can be found in Tenenhaus et al. [5]. In the PLSPM used for the GBPLS1 algorithm, genotype information from SNPs within the same gene are combined into a single LV (i.e., gene score) by constructing a linear function of the SNPs in , (*i* = 1, 2, …, 100). The resulting coefficients of the SNPs are the outer weights/coefficients.

In the second stage of the GBPLS1 approach, the inner model coefficients are estimated by ordinary least squares in the multiple regression model given by:(1)

for *j* = 1, 2, where *Z_i_* is the LV for gene *i*. We then order the absolute values of the inner model coefficients for the gene scores (i.e., *β_i_*, *i* = 1, 2, …, *t*) to identify the *q* most important ones in explaining the trait *Q_j_*, where *q* in the analysis is taken to be 25, 35, and 50. To determine the relative importance of the SNPs in the construction of the *q* most important gene scores, we ordered the absolute values of the outer weights and recorded the corresponding ranks (called SNP ranks) for each SNP.

Although the GBPLS1 approach can deal with a dimension corresponding to 100 genes, it has computational problems when we run it for more than 100 genes because the algorithm simply uses the least-squares estimator that would fail with large dimensionality. To remedy this problem, we propose a similar algorithm, GBPLS2, that incorporates a partial least-squares and penalized regression for calculating the outer and inner weights, respectively, to handle higher dimensions.

Partial least-squares regressions aim to derive the orthogonal latent components using the cross-covariance matrix between the response variable and the explanatory variables [6]. We calculate outer weights and gene scores *T_i_* by solving the following maximization problem:(2)

where *y* is the trait and the gene score is taken to be the projection of  on the outer coefficient vector  , that is,(3)

for *t* = 100, 250, 500, 750 and *i* =1, 2, …, *t*.

In the second stage, we apply the LASSO (least absolute shrinkage and selection operator) penalty [7] to implement regression analysis of traits on the gene scores *T_i_* and other non-SNP covariates and the first 10 PCs in which only gene scores were penalized. The penalty parameter was determined for each replicate by 10-fold cross-validation. The genes with nonzero inner coefficients and the rankings of the corresponding outer coefficients for the SNPs within these genes are the outcomes of the GBPLS2 algorithm.

We carried out our GBPLS analyses using R packages plspm (the factor scheme), glmnet, and plsgenomics, which were downloaded from [http://cran.r-project.org/].

## Results

**Figure 2:**
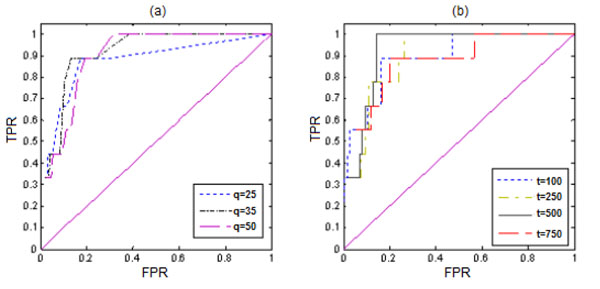
**Receiver operating characteristic curves for trait Q_1_ using (a) GBPLS1 and (b) GBPLS2.** TPR (or FPR) is the proportion of significant genes that are among true associated (or true unassociated) genes in at least *c* replications (*c* = 1, 2, 3, …, 25).

The GBPLS1 and GBPLS2 algorithms were applied to the 200 replicates. For a given cutoff value *c*, a gene is said to be associated if it is selected in at least *c* of the 200 replicates. For each method, we calculated the true-positive rate and the false-positive rate (TPR and FPR, respectively) by setting *c* = 1, 2, …, 25. Figure 2 shows the receiver operating characteristic (ROC) curves for the trait Q_1_ using GBPLS1 for *q* = 25, 35, 50 (Figure 2a) and GBPLS2 for *t* = 100, 250, 500, 750 (Figure 2b). The ROC curves for Q_2_ are similar, and we omit the detailed results for brevity. In general, FPRs for Q_2_ are generally lower than those for Q_1_. Based on these plots, *q* = 35 and *t* = 500 seem to be better choices for Q_1_, whereas *q* = 25 and *t* = 750 fit slightly better for Q_2_. The results that represent the optimal choice of *q*, *t*, and *c* for each method and trait are given in Table 1. The GBPLS2 approach can detect all the genes correctly with a 15% FPR for Q_1_, whereas the GBPLS1 approach has a lower FPR but also a lower TPR. The performances of the GBPLS1 and GBPLS2 approaches are comparable for Q_2_, with GBPLS2 yielding a slightly smaller FPR.

**Table 1:** False-positive and true-positive rates for the GBPLS1 and GBPLS2 methods for selected values of *c*, *t*, and *q*

Trait	GBPLS1		GBPLS2	
	
	TPR	FPR	TPR	FPR
Q_1_	0.89	0.13	1	0.15
Q_2_	0.85	0.12	0.85	0.10

We also include the results for a SNP-based approach to demonstrate the advantages of the gene-based approach. We applied the *t*-test by treating SNPs as the two group variables because there are no homozygous genotypes for the minor alleles. We calculated *p*-values for each replicate and calculated the median of the *p*-values for traits Q_1_ and Q_2_. This SNP-based analysis indicated that no variants (except C13S522 and C13S523 in *FLT1*) exhibited genome-wide significance (<2 × 10^−6^) with Bonferroni correction.

The rest of the analysis is run only for the methods summarized in Table 1. The median of the SNP ranks are calculated for each gene among the replicates for which that particular gene has been selected. If a SNP is among the top half based on the calculated ranks, then the SNP is said to be associated. The results for the set of genes and associated SNPs of the simulation model for Q_1_ are summarized in Table 2. The results indicate that both methods can capture some of the important SNPs with small MAF, although the GBPLS1 approach seems to be slightly better than the GBPLS2 approach in terms of detecting variants with extremely small MAFs (= 0.000717). The results for Q_2_ are similar.

**Table 2:** Summary of results for Q_1_

Gene	GBPLS1 SNPs	GBPLS2 SNPs
*ARNT*	C1S6533, C1S6542, **C1S6561**	C1S6533, C1S6540, C1S6542
*ELAVL4*	**C13181**	None
*FLT1*	C13S431, C13S522, C13S523, C13S524	C13S431, C13S522, C13S523, C13S524
*FLT4*	C5S5133, **C5S5156**	C5S5133
*HIF1A*	C14S1729, C14S1734	C14S1729, C14S1734
*HIF3A*	N/A	None
*KDR*	**C4S1873**, **C4S1877**, C4S1878, C4S1884, **C4S1889**	C4S1861, **C4S1877**, C4S1878, C4S1884, **C4S1889**
*VEGFA*	C6S2981	C6S2981
*VEGFC*	**C4S4935**	**C4S4935**

Listed in the first column are the genes that are associated with Q_1_ in the simulation model. “N/A” means that the gene is not detected, and “None” means that none of the causal SNPs are detected even though the gene is detected by the corresponding method. The SNP names in bold are those with MAF = 0.000717.

## Discussion and conclusions

In this paper, we used the 1000 Genomes Project second-generation sequencing genotype data in conjunction with simulated phenotypic data made available through GAW17 to demonstrate our gene-based approach for detecting associations between common diseases and rare variants. Our results are encouraging because some of the causal SNPs in the simulation model were successfully detected, whereas they would have remained elusive with a SNP-based method. However, because the SNP genotypes were held fixed in the simulated replicates, further evaluation is warranted to fully assess the effectiveness of the methods under more general and more diverse settings.

It is also worth pointing out that, although population substructure is not part of the feature of the simulation model, our methods are capable of accommodating such a complex scenario. Our results indicate that statistical power was not greatly affected by the inclusion of factors to account for potential stratification caused by population substructure. However, this assessment is preliminary and, as such, further investigation is needed for more comprehensive evaluation. Moreover, the effectiveness of the methods for correctly accounting for population structure will need to be carefully evaluated.

Finally, we note that our analysis and summary of the results are with knowledge of the answers. Nevertheless, the proposed approach was not developed based on the specific simulation model; rather, it was designed to detect variants, including rare ones, that work together within the same genes for causing or regulating disease phenotypes. Thus we expect our method to be effective in real data analysis.

## Competing interests

The authors declare that they have no competing interests.

## Authors’ contributions

AST and SL conceived the project, designed the algorithm and wrote the manuscript. AST implemented the algorithm and analyzed the data. SL supervised the research and polished the manuscript. Both authors read and approved the final manuscript.
